# N-3 Fatty Acid Rich Triglyceride Emulsions Are Neuroprotective after Cerebral Hypoxic-Ischemic Injury in Neonatal Mice

**DOI:** 10.1371/journal.pone.0056233

**Published:** 2013-02-20

**Authors:** Jill J. Williams, Korapat Mayurasakorn, Susan J. Vannucci, Christopher Mastropietro, Nicolas G. Bazan, Vadim S. Ten, Richard J. Deckelbaum

**Affiliations:** 1 Institute of Human Nutrition, Department of Pediatrics, College of Physicians and Surgeons of Columbia University, New York, New York, United States of America; 2 Department of Pediatrics, Weill Cornell Medical College of Cornell University, New York, New York, United States of America; 3 Department of Pediatrics, Children’s Hospital of Michigan and Wayne State University, Michigan, United States of America; 4 Neuroscience Center of Excellence, Louisiana State University Health Sciences Center, New Orleans, Louisiana, United States of America; 5 Department of Pediatrics, College of Physicians and Surgeons of Columbia University, New York, New York, United States of America; University of South Florida, United States of America

## Abstract

We questioned if acute administration of n-3 fatty acids (FA) carried in n-3 rich triglyceride (TG) emulsions provides neuroprotection in neonatal mice subjected to hypoxic-ischemic (H/I) brain injury. We examined specificity of FA, optimal doses, and therapeutic windows for neuroprotection after H/I. H/I insult was induced in C57BL/6J 10-day-old mice by right carotid artery ligation followed by exposure to 8% O_2_ for 15 minutes at 37°C. Intraperitoneal injection with n-3-rich TG emulsions, n-6 rich TG emulsions or saline for control was administered at different time points before and/or after H/I. In separate experiments, dose responses were determined with TG containing only docosahexaenoic acid (Tri-DHA) or eicosapentaenoic acid (Tri-EPA) with a range of 0.1–0.375 g n-3 TG/kg, administered immediately after H/I insult. Infarct volume and cerebral blood flow (CBF) were measured. Treatment with n-3 TG emulsions both before- and after- H/I significantly reduced total infarct volume by a mean of 43% when administered 90 min prior to H/I and by 47% when administered immediately after H/I. In post-H/I experiments Tri-DHA, but not Tri-EPA exhibited neuroprotective effects with both low and high doses (*p*<0.05). Moreover, delayed post-H/I treatment with Tri-DHA significantly decreased total infarct volume by a mean of 51% when administered at 0 hr, by 46% at 1 hr, and by 51% at 2 hr after H/I insult. No protective effect occurred with Tri-DHA injection at 4 hr after H/I. There were no n-3 TG related differences in CBF. A significant reduction in brain tissue death was maintained after Tri-DHA injection at 8 wk after the initial brain injury. Thus, n-3 TG, specifically containing DHA, is protective against H/I induced brain infarction when administered up to 2 hr after H/I injury. Acute administration of TG-rich DHA may prove effective for treatment of stroke in humans.

## Introduction

Stroke is the third major cause of death in adults in the United States, after coronary heart disease and cancer. It is estimated that in the USA stroke-related medical costs and disability were approximately $73.7 billion in 2010 [1]. In addition, with an estimated incidence of 1 in 2300 to 5000 births, stroke is more likely to occur in the perinatal period than at other times in childhood [2]. Ischemic stroke in neonates is a disorder associated with significant long-term neurologic morbidity [2]. Twenty to 60% of survivors exhibit long-term detrimental neuropsychological consequences which include mental retardation, cerebral palsy, and behavioral disorders [2].

Multiple mechanisms are associated with neuronal damage in ischemic stroke. Abnormalities of cerebral blood flow (CBF) can result in metabolic responses such as increased anaerobic metabolism, and biochemical responses such as increased excitatory amino acids, intracellular accumulation of calcium, activation of nitric oxide synthesis, and production of free radicals. These mechanisms occur over a range of time, with early events within minutes of energy loss, and then progress after hours and days following the insult culminating in inflammatory events, cell injury and tissue death [3]. Some of these mechanisms could be targets for neuroprotective therapies aimed to prevent neuronal damage [4].

n-3 triglycerides (TG) are studied here as a potential stroke treatment as there are multiple cellular and metabolic functions that are modulated by n-3 fatty acids (FA), especially docosahexaenoic acid (DHA), eicosapentaenoic acid (EPA), and their biologically active derivatives that could play a role in neuroprotection from ischemic stroke [5]. n-3 FA are precursors to lipid mediators that play an important role in regulating multiple pathways including regulation of cytokines, chemokines, and pro-inflammatory signaling molecules [6]. n-3 FA are anti-inflammatory, anti-apoptotic, and they decrease coagulation factors and vascular resistance [6]. They also bind to transcription factors affecting the regulation of genes [7]. These effects may contribute to many “positive” roles that n-3 FA may play in human health, including those in the areas of immune/inflammatory outcomes, neurological degeneration, and cardiovascular disease [8].

Ischemic stroke triggers activation of phospholipase A2 (PLA_2_) which stimulates the release of esterified FA from brain membranes [9], and results in altering the FA content of specific brain membrane phospholipid pools to play a role in tissue injury [9]. In addition, released FA are enzymatically converted to anti-inflammatory lipid messengers. For example, DHA is converted to neuroprotectin D1 (NPD1) [9] which exhibits potent neuroprotective bioactivity after ischemic injury via protection from oxidative stress induced apoptosis and anti-inflammatory pathways. However, when the ischemic insult is severe, endogenous protection mechanisms are not enough to protect the brain from inflammation and the ensuing damaging pathways [10]. Exogenous administration (continuous infusion) of DHA bound to human albumin has been previously shown to be neuroprotective in an adult rodent stroke model [11]. However, albumin at high dose expands intravascular volume and may precipitate congestive heart failure, but with concomitant NPD1 synthesis in the ipsilateral side after stroke [11]. Also, direct infusion of free FA at high doses can lead to encephalopathy and hepatotoxicity [12]. Thus, in the present study we utilized acute administration of n-3 rich TG emulsions that allows for slower release of n-3 FA [13], [14].

The brain’s response to hypoxia-ischemia or stroke is age-dependent both in the degree and timing of apoptosis and in the inflammatory response [15], [16]. We have studied hypoxic-ischemic (H/I) brain injury in neonatal mice using an H/I model well established in our laboratories [17]. Our results demonstrate that, in developing H/I brain, acute administration of n-3 TG up to 2 hr after H/I insult is protective against H/I-induced brain injury. Specifically, tridocosahexaenoic acid (Tri-DHA), but not trieicosapentaenoic acid (Tri-EPA), has a major role in early neuroprotection after acute H/I brain injury.

## Materials and Methods

### Ethics Statement

All research studies were carried out according to protocols approved by the Columbia University Institutional Animal Care and Use Committee (IACUC) and in accordance with the Association for Assessment and Accreditation of Laboratory Animal Care guidelines.

### Materials

Tri-DHA and Tri-EPA were purchased from Nu-Chek Prep, Inc. (Elysian, MN). Egg yolk phosphatidylcholine was obtained from Avanti Polar-Lipids, Inc. (Alabaster, AL).

### Lipid Emulsions

Four different types of lipid emulsions were used in our experiments. n-3 fish oil-based and n-6 soy oil-based emulsions were commercially prepared intravenous phospholipid-stabilized emulsions, and n-3 TG contained high concentrations of n-3 FA as previously described (Table S1) [13], [18]. In the text and Figure Legends these are referred to as n-3 TG and n-6 TG emulsions. The n-6 TG emulsions were produced from soy bean oil rich in n-6 FA: linoleic acid constituting about 55% of total FA. In contrast, the n-3 TG emulsions, which contained fish oil, were rich in EPA (up to 28%) and DHA (up to 30%). For doses of injected n-3 TG emulsions, we calculated that the amount adiministered contains 50% of the TG–FA as DHA and EPA (Table S1). Thus, 1 gm of TG emulsions is expressed as 0.5 gm n-3 TG.

The pure Tri-DHA and Tri-EPA emulsions were VLDL-sized and laboratory-made with TG oil and egg yolk phospholipid using sonication and centrifugation procedures that we have previously detailed [19]–[21]. Briefly, 200 mg Tri-DHA (Tri-DHA oil >99%) or Tri-EPA (Tri-EPA oil >99%) was mixed with a 5∶1 weight ratio of egg yolk phosphatidylcholine (40 mg). The mixture was fully evaporated under N_2_ gas, and was further desiccated under vacuum overnight at 4°C. The dried lipids were resuspended in 1 mL of lipoprotein-free buffer (LPB) (150 mmol/L NaCl, 0.5 ml of 0.1% glycerol and 0.24 mmol/L EDTA, pH 8.4, density 1.006 g/mL) at 60°C with added sucrose (100 mg/1 mL LPB) to remove excess phospholipid liposomes. The lipid emulsions were then sonicated for 1 hr at 50°C, 140 W under a stream of N_2_ using a Branson Sonifier model 450 (Branson Scientific, Melville, NY). After sonication, the solution was dialyzed in LPB for 24 hr at 4°C to remove sucrose. The final emulsions comprising VLDL-sized particles were analyzed for the amount of TG and PL by enzymatic procedure using GPO-HMMPS, glycerol blanking method (Wako Chemicals USA, Inc., Richmond, VA) and choline oxidase-DAOS method (Wako Chemicals USA, Inc., Richmond, VA). The TG: phospholipid mass ratio was 5.0±1.0∶1 similar to that of VLDL-sized particles. The emulsions were then stored under argon at 4°C and were used for experiments within 2 weeks of preparation.

### Unilateral Cerebral H/I

Three-day-old C57BL/6J neonatal mice of both genders were purchased from Jackson Laboratories (Bar Harbor, ME) with their birth mother. We used the Rice-Vannucci model of H/I modified to p10 neonatal mice [17]. Briefly, on postnatal day 10 H/I was induced by the ligation of the right common carotid artery, which was further cauterized and cut under isoflurane anesthesia. The person who performed the procedure was blinded to the lipid emulsion treatment during the surgery and after the surgery. The entire surgical procedure was completed within 5 min for each mouse. Pups were then allowed to recover with their dams for 1.5 hr. Surrounding temperature during experiments was kept at 28°C. Mice were then exposed to systemic hypoxia for 15 min in a hypoxic chamber in a neonatal isolette (humidified 8% oxygen/nitrogen, Tech Air Inc., White Plains, NY) [17]. The ambient temperature inside the chamber during hypoxia was stabilized at 37±0.3°C. To minimize a temperature-related variability in the extent of the brain damage, during the initial 15 hr of reperfusion mice were kept in an isolette at the ambient temperature of 32°C.

### Quantification of Brain Infarction

After 24 hr of reperfusion, the animals were sacrificed by decapitation and brains were immediately harvested. 1-mm coronal slices were cut by using a brain slicer matrix. Slices were then immersed in a PBS solution containing 2% triphenyl-tetrazolium chloride (TTC) at 37°C for 25 min. TTC is taken up into living mitochondria, which converts it to a red color [22]. Thus, viable tissue stains brick-red, and nonviable (infarcted) tissue can be identified by the absence of staining (white). Using Adobe Photoshop and NIH Image J imaging applications, planar areas of infarction on serial sections were summed to obtain the volume (mm^3^) of infarcted tissue, which was divided by the total (infarcted+non-infarcted) volume of the hemisphere ipsilateral to carotid artery ligation, and expressed as a percentage of total volume.

### Experimental Groups

H/I brain injury was induced in different groups of animals, which received specific treatments before and after H/I injury. Animals followed the following treatment protocols.

- Pre-H/I treatment of n-3 TG (containing both DHA and EPA) or n-6 TG emulsions. Two doses of n-3 TG or n-6 TG emulsions or vehicle (saline, equal volumes/kg) were administered to non-fasting rodents at a fixed dose of 3 mg of n-3 or n-6 TG-FA per mouse for each injection (equivalent to a maximum of 1.5 g of total TG/kg; p10 mice weighed 4–6 gm for these experiments). The first dose was intraperitoneally (i.p.) administered immediately after surgery, and the second immediately at the end of the 15 min hypoxic period. Volumes injected for TG emulsions and saline were always equal.

Since n-3 emulsions contain low concentrations of alpha-tocopherol as an anti-oxidant agent, in separate experiments an equivalent dose of pure alpha-tocopherol to match the content of n-3 emulsion content (0.8 g/L) was given to neonatal mice by i.p. injection of alpha-tocopherol (Vital E®, Intervet, Schering Plough) at a dose of 5 mg alpha-tocopherol/kg body weight, the amount contained in each i.p. injection of the n-3 TG emulsions.

- **Post-H/I treatment of n-3 TG (containing both DHA and EPA).** Two doses of the commercially available n-3 TG emulsion or saline were i.p. injected into non-fasting rodents at 0.75 g of n-3 TG/kg body weight for each dose (equivalent to 1.5 g of total TG/kg). The first dose was administered immediately after 15-min hypoxia, and the second at 1 hr after start of the reperfusion period.
- **Dose response, timing and specificity of n-3 TG.** Two types of n-3 containing lipid emulsions either Tri-DHA or Tri-EPA (0.1 g n-3 TG/kg or 0.375 g n-3 TG/kg body weight for each dose) were administered twice to non-fasting rodents (according to the amount of DHA and EPA in the mixed n-3 TG emulsions) (Table S1). The first dose was initially administered immediately after 15-min hypoxia, and the second after 1 hr of reperfusion. Then in different sets of experiments, we determined the efficacy of Tri-DHA emulsions, with the initial injection administered at four-time points (0 hr, or at 1-hr, 2-hr or 4-hr after H/I), 0.375 g n-3 TG/kg body weight for each dose. For the immediate treatment of 0 hr, the first dose was injected immediately after 15-min hypoxia, with a second injection after 1 hr of reperfusion, whereas in the “delayed” treatments, the first dose was given after the 1^st^ or 2^nd^ or 4^th^ hr of reperfusion and a second dose was administered 1 hr after the 1^st^ dose.

### Blood TG and Glucose Levels

Blood samples for blood TG were directly taken from left ventricle of hearts under isoflurane inhalation from a separate cohort of non-fasting, 10-day-old mice. Samples were taken over a 5 hr period after a single i.p. injection of either 0.75 g n-3 TG/kg commercially available n-3 rich TG (DHA and EPA) emulsions or saline. Total plasma TG was enzymatically measured by GPO-HMMPS, glycerol blanking method (Wako Chemicals USA, Inc., Richmond, VA). For glucose levels, blood samples were taken from mouse tails from a separate cohort of non-fasting10-day-old mice. Samples were taken at two time points from each mouse. The first sample was taken at time zero before surgery and TG injection, and the second at about 10 min after H/I and TG injection (approximately 100 min after surgery as described under the Unilateral Cerebral H/I protocol above). Blood glucose levels were electrochemically measured in mg/dL by a glucose meter (OneTouch Ultra, LifeScan, Inc., Milpitas, CA).

### Cerebral Blood Flow (CBF) by Laser Doppler Flowmetry (LDF)

In a cohort of neonatal C57BL/6J mice pups subjected to carotid artery ligation and recovery as described above, relative CBF was measured during hypoxia in ipsilateral (right) hemispheres using a laser Doppler flowmeter (Periflux 5000). In these mice, in preparation for CBF measurement the scalp was dissected under isoflurane anesthesia and Doppler probes were attached to the skull (2 mm posterior and 2 mm lateral to the bregma) using fiber optic extensions. Only local anesthesia (1% lidocaine) was used postoperatively. Mice were then placed into a hypoxia chamber (8%O_2_/92%N_2_). Changes in CBF in response to hypoxia were recorded for 20 min and expressed as percentage of the pre-hypoxia level for n-3 treated and saline treated neonatal mice.

### Bleeding Time after n-3 TG Injection

Bleeding times were measured in mice after severing a 3-mm segment of the tail (as previously described) [23]. We gave two doses of saline vs. n-3 TG in a similar time frame as the original protocol: an initial injection followed by a second injection at 2 hr later. Bleeding times were measured at 45 min after the second dose. The amputated tail was immersed in 0.9% isotonic saline at 37°C, and the time required for the stream of blood to stop was defined as the bleeding time. If no cessation of bleeding occurred after 10 min, the tail was cauterized and 600 s was recorded as the bleeding time.

### Long-term Assessment of Brain Tissue Death

A long-term assessment of cerebral injury was performed at 8 wk after neonatal H/I insult. This cohort of mice at p10 underwent unilateral H/I followed by post H/I injections with either 0.375 g Tri-DHA/kg (n = 6) or saline (n = 5) as described above. At 8 wk after H/I mice were sacrificed by decapitation. Brains were removed, and embeded in *Tissue Tek*-*OTC*-compound (Sakura Fineteck, Torrance, CA) with subsequent snap freezing in dry ice-chilled isopentane (−30°C), and stored at −80°C. For analysis, coronal sections (10 µm every 500 µm) were cut serially in a Leica cryostat and mounted on Superfrost slides (Thermo Scientific, Illinois). Sections were processed for Nissl staining by using Cresyl Violet Acetate (Sigma-Aldrich, St. Louis, MO). Using Adobe Photoshop and NIH Image J imaging applications, 9 sections from each brain containing both the right and left hemispheres were traced for brain tissue area. As previously described [24] the area of left control or contralateral hemisphere which had not had injury was given a value in 100% for each animal. The brain area remaining in the right injured ipsilateral hemisphere was then compared to the left hemisphere, and the difference was taken as the percent right brain tissue loss, for each animal.

### Statistical Analyses

Data are presented as mean ± SEM. We compared plasma TG levels at each time point after i.p. injection of n-3 TG emulsion. Student *t* tests were used for 2-group comparisons. 1-way ANOVA, followed by Bonferroni procedure for post hoc analysis to correct for multiple comparisons, was used to compare the differences among the emulsions on the infarct areas across coronal sections. Statistical significance, which was analyzed by using SPSS software 16.0 (SPSS Inc., Chicago, IL), was determined at *p*<0.05.

## Results

### Effects of n-3 TG on Blood Triglyceride and Glucose Levels, and Bleeding Time

**Figure 1:**
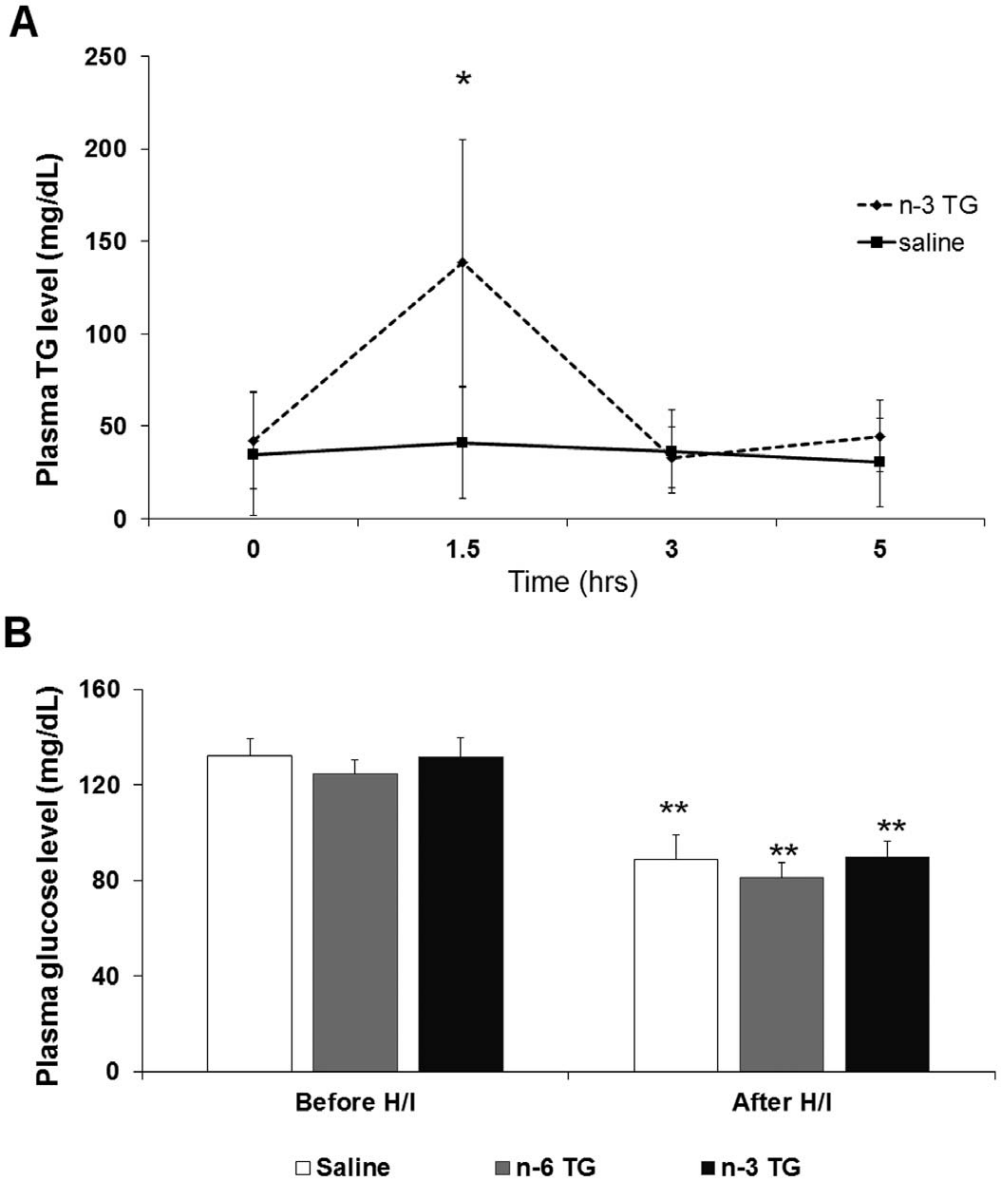
Blood TG and glucose levels after TG emulsion injections. A. Total plasma TG concentrations (mg/dL) in non-fasting neonatal mice (p10), acutely injected (i.p.) with saline or n-3 TG emulsion (0.75 g n-3 TG/kg body weight). *p<0.05 (n = 3–8 in each group). Each data point represents the mean ± SEM of 3 separate experiments. B. Plasma glucose concentrations (mg/dL) in non-fasting mice (p10) in post-H/I treatment of n-3 TG or n-6 TG or vehicle (saline) comparing to the time between before H/I and after H/I. **p<0.001 (n = 5–9 in each group).

To determine if TG from the n-3 TG emulsions were systemically absorbed, we examined the blood TG levels up to 5 hr after i.p. injection. After n-3 TG injection, there was a substantial increase of TG levels up to three fold higher at 1.5 hr (*p*<0.05) compared to the baseline, followed by a decrease of levels to baseline at 3 and 5 hr (Figure 1A). This indicates that n-3 TG had entered into the blood stream and were being catabolized. In comparison, TG levels of saline-injected mice remained constant over the 5 hr time period reflecting normal blood TG levels in neonates.

After H/I, blood glucose levels might affect infarct size [25]. Therefore, we measured blood glucose levels in each group (n-3 TG vs. n-6 TG vs. saline control) prior to surgery and after 15-min H/I after TG or saline injection (Figure 1B). No difference in blood glucose levels among groups was observed when comparing at the same time point. Still, after H/I insult, blood glucose levels decreased similarly, about 30% or more, in all groups (*p*<0.05).

There was no difference in capillary bleeding times in n-3 treated mice (437±82 sec) as compared to saline controls (418±90 sec).

### n-3 TG does not Change Cerebral Blood Flow after H/I

**Figure 2:**
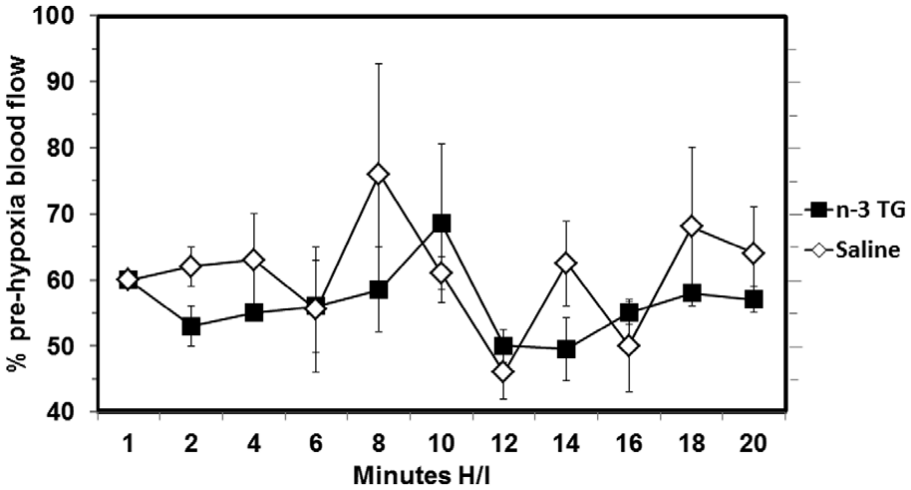
n-3 TG injection and cerebral blood flow after H/I. Cerebral blood flow (CBF) was measured by laser Doppler flowmetry (LDF) in neonatal mice after carotid artery ligation. Relative CBF was measured every two minutes during hypoxia in ipsilateral (right) hemispheres using a laser Doppler flowmeter. Changes in CBF in response to hypoxia were recorded for 20 min and expressed as percentage of the pre-hypoxia level for n-3 TG treated (n = 3) and saline treated (n = 5) neonatal mice.

There was no effect on CBF in the ipsilateral hemisphere of n-3 treated neonatal mice as compared to saline treated animals. Immediately after right common carotid artery ligation, and initiation of hypoxia at 8% oxygen, CBF was approximately 25% of initial (pre-H/I) level in the ipsilateral hemisphere in both control and n-3 treated groups, and this was maintained for the duration of hypoxia. In the contralateral (unligated) hemisphere blood flow was unchanged in both groups. Very similar blood flow levels were maintained in neonatal H/I mice whether they were saline treated or n-3 treated in this model (Figure 2).

### n-3 TG but not n-6 TG Protects Brain against H/I Injury

**Figure 3:**
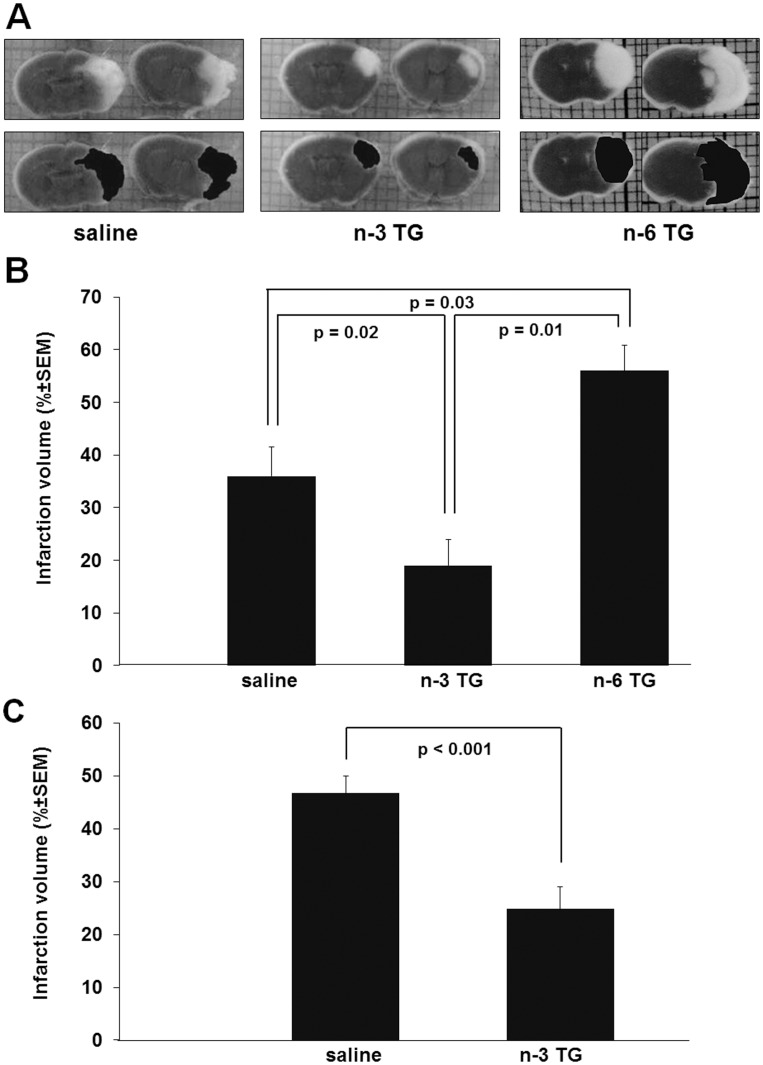
TTC stained coronal sections of mouse brain and quantification of injury after H/I. A. TTC-stained coronal sections of representative mouse brains from saline treated, n-3 TG treated and n-6 TG treated. The top panel shows images of coronal mouse brain that are sliced and then stained with TTC (grey for living tissue and white for the infarcted tissue), and the lower panel shows the infarcted areas that are traced in black for quantification. B. Percent of cerebral infarct volume from pre-H/I mice treated with n-3 TG emulsion (n = 28) or n-6 TG emulsion (n = 10) or saline control (n = 27). C. Percent of cerebral infarct volume after H/I in the post-H/I treatment protocol in mice treated with n-3 TG emulsion (n = 18) or saline control (n = 18). Each bar represents the mean ± SEM of 5–7 independent experiments.

Coronal sections of brains were stained with TTC to quantify the extent of post H/I brain injury and the effect of n-3 TG injection (Figure 3). Figure 3A shows representative images of neonatal mouse brain from saline treated, n-6 TG emulsion treated and n-3 TG emulsion treated mice with pre-and-post injection after H/I, respectively. In all H/I animals, tissue death was localized to the right hemisphere (ipsilateral to ligation) as illustrated by the white areas in the upper panels of Figure 3A. The image in the lower panels, Figure 3A, demonstrates tracings of the infarcted areas for quantifying infarct volume using NIH Image J. The brains from saline treated animals exhibited a consistent pannecrotic lesion involving both cortical and subcortical regions ipsilateral to the ligation. In the majority of the animals the neuroprotection after n-3 TG injection was most marked in the subcortical area, whereas saline treated mice had large cortical and subcortical infarcts (Figure 3A).

Infarct volume was substantially decreased in n-3 TG treated mice (n = 28) compared to saline treated littermates (control) (n = 27), 19.9±4.4% vs. 35.1±5.1%, respectively (p = 0.02) (Figure 3B). There was a significant increase in infarct volume with n-6 TG emulsion injection compared to saline control (p = 0.03) and the n-3 TG groups (p<0.01).

Because alpha-tocopherol is a component of the TG emulsions (present in low concentrations to prevent FA oxidation) we also used TTC staining to compare the extent of cerebral H/I injury in alpha-tocopherol treated and saline treated neonatal mice. There was no significant difference in infarct volume between brains in alpha-tocopherol injected mice compared to saline treated mice (data not shown).

We next determined if n-3 TG are effective if injected only after H/I (without injection prior to H/I (Figure 3C)). Similarly, the smaller n-3 TG associated lesions were mainly subcortical (data not shown). Compared to saline controls in the immediate post-H/I treatment the total infarct area was significantly reduced almost 50% in the n-3 TG post H/I treated group.

### DHA but not EPA is Neuroprotective after H/I

**Figure 4:**
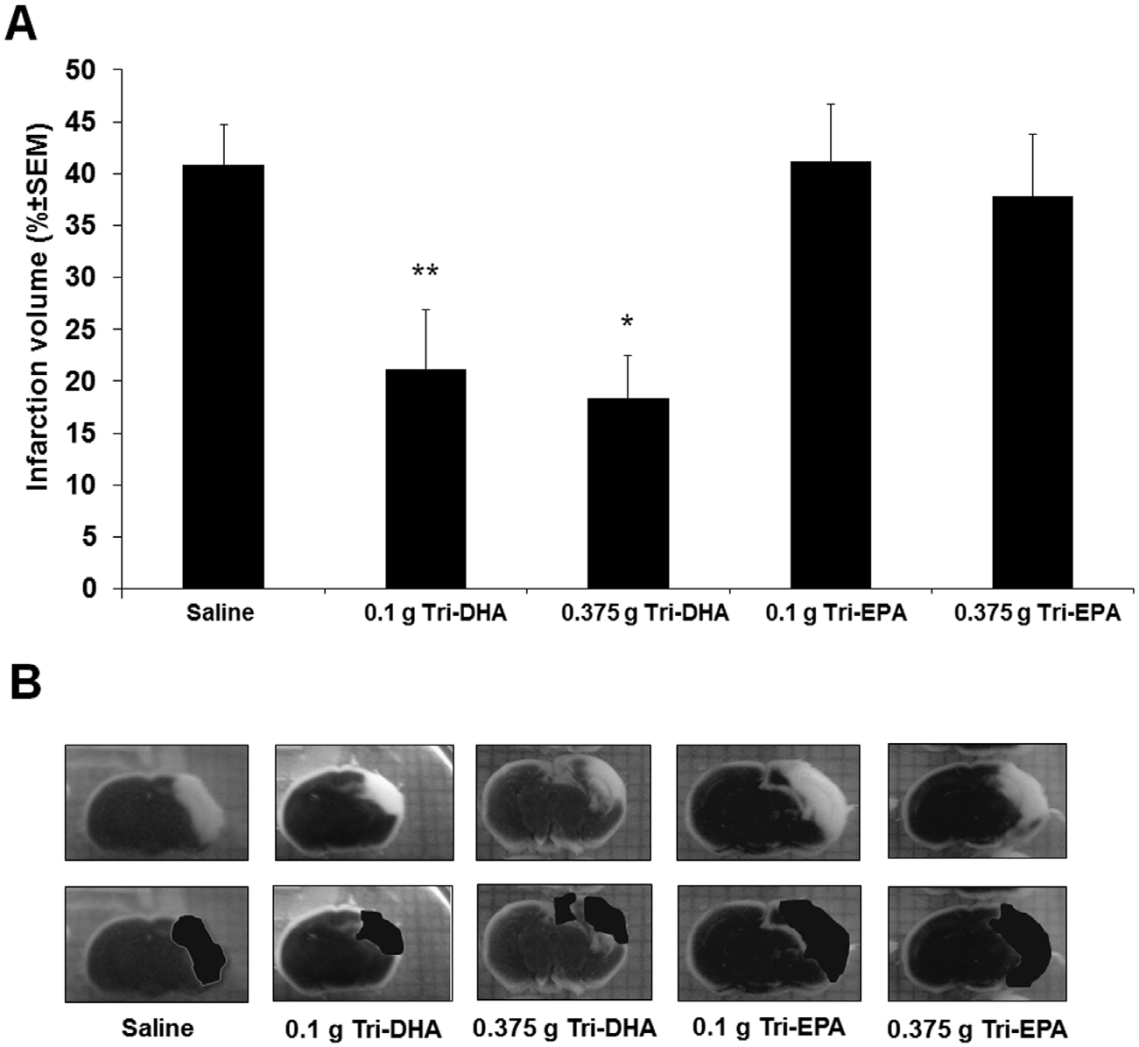
Effect of Tri-DHA versus Tri-EPA on cerebral infarct volume after H/I. A. Mice were subjected to 15 min ischemia followed by 24-hr reperfusion and received 2 i.p. administrations (immediately after ischemia and 1 hr of reperfusion) at 2 doses (0.1 g n-3 TG/kg and 0.375 g n-3 TG/kg). Each bar represents the mean ± SEM of 5–7 independent experiments performed using the same H/I model. B. TTC-stained coronal sections of representative mouse brains from saline treated, 0.1 g Tri-DHA, 0.375 g Tri-DHA, 0.1 g Tri-EPA and 0.375 g Tri-EPA. * *p*<0.05 compared to other groups except 0.1 g Tri-DHA/kg. ** *p*<0.05 compared to other groups except 0.375 g Tri-DHA/kg and 0.375 g Tri-EPA/kg.

To determine possible differences in neuroprotection of EPA vs. DHA, we studied the extent of brain injury using the post-H/I treatment protocol with Tri-DHA vs. Tri-EPA in two dosages (0.1 g TG/kg vs. 0.375 g TG/kg). No statistical differences in brain infarct volume between 0.1 g TG/kg and 0.375 g TG/kg Tri-DHA treated groups were observed. However, compared to saline control, total infarct size was reduced by a mean of 48% and 55% by treatment with 0.1 and 0.375 g TG/kg Tri-DHA, respectively (Figure 4). Neuroprotection was not observed with Tri-EPA injection at either of the two doses compared with saline treatment.

**Figure 5:**
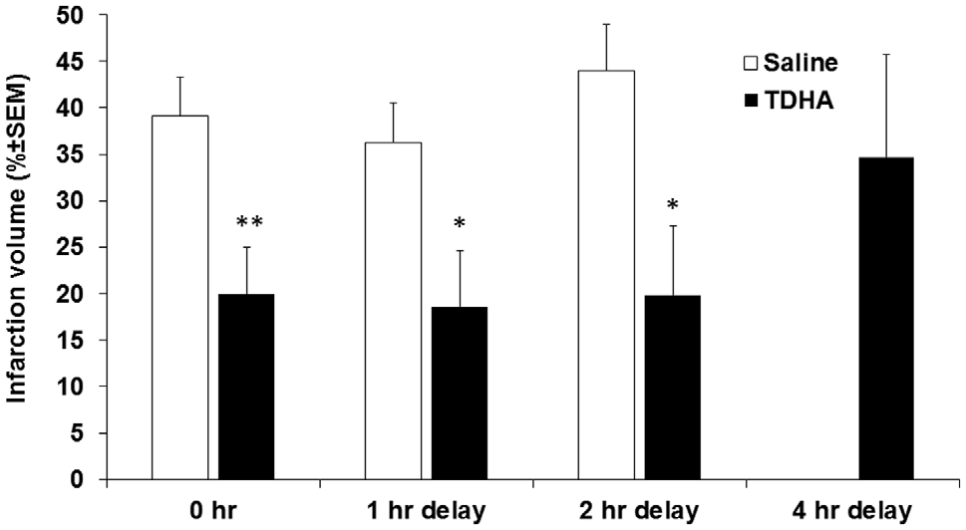
Effects of delayed treatment with Tri-DHA on cerebral infarct volume after H/I. Mice were subjected to 15-min ischemia followed by 24-hr reperfusion and received 2 i.p. administrations at four-time points (immediate [0,1 hr], delayed 1-hr [1,2 hr], or 2-hr [2,3 hr] or 4-hr [4,5 hr] treatments). Each bar represents the mean ± SEM of 5–7 independent experiments. * *p*<0.05; ** *p*<0.001 vs. saline control (n = 10–20 in each group).

To better approximate realistic timelines for neuroprotection after stroke for humans we performed delayed treatment protocols to study the therapeutic window of Tri-DHA emulsions. No protective effect from Tri-DHA after a 4-hr delay in treatment was noted compared with saline group. However, Tri-DHA administered at 0 hr immediately post H/I, and then delayed 1-hr and 2-hr post stroke showed similar reduced (∼ 50%) brain infarct volumes compared to saline treated animals (Figure 5). This substantial protection occurred mainly in subcortical areas similar to the findings described above.

### Long-term Neuroprotection

**Figure 6:**
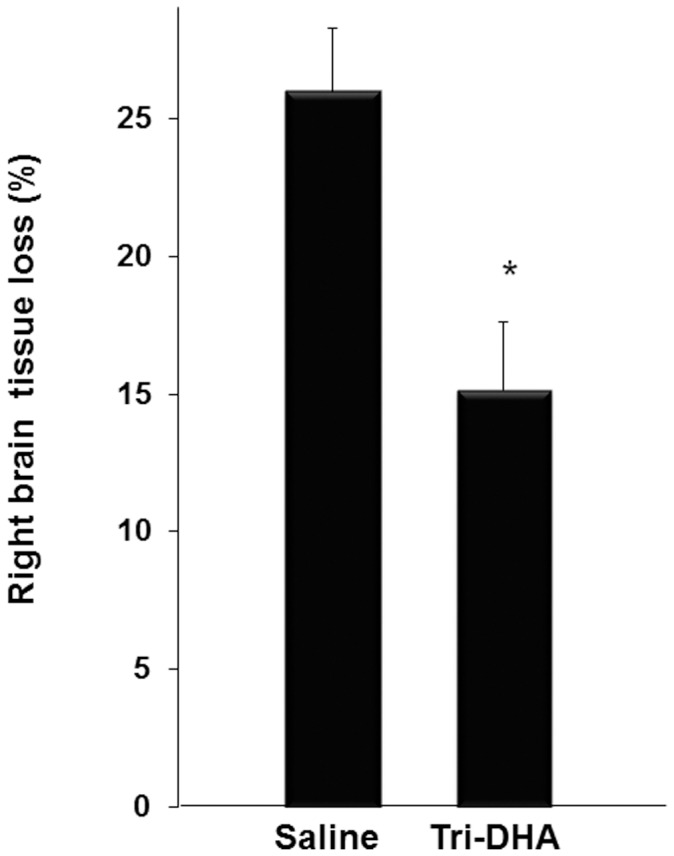
Long-term effect of Tri-DHA on cerebral tissue death at 8 wk after H/I. Mice were subjected to 15-min H/I and received 2 i.p. administrations of 0.375 g Tri-DHA/kg (n = 6) vs. saline (n = 5). At 8 wk after H/I mice were sacrificed and brains were fixed with 4% paraformaldehyde and 10 µm-thick slices were cut and preserved. Nissl staining was used for identifying neuronal and brain structure. As described in Methods right brain tissue loss in relation to the contralateral hemisphere was calculated and expressed as a percentage. Each bar represents the mean ± SEM. * *p*<0.05.

Coronal brain sections of adult mice were processed for Nissl staining (Figure 6) to examine the effects of H/I and Tri-DHA treatment on brain and neuronal cell loss for long-term outcome at 8 wk after H/I insult. As compared to the left control (contralateral hemisphere), the injured areas of the right hemisphere display gross neuronal cell loss. As shown in Figure 6, brain tissue loss was markedly increased by 1.67 fold in the right hemisphere of saline-treated mice (n = 5) as compared to Tri-DHA treated mice (n = 6), 25.0±2.4% vs. 15.0±2.5%, respectively (p = 0.02). Thus, neuroprotection after injury and Tri-DHA injection that are observed 24 hr after H/I can be demonstrated histologically almost 2 months after the initial stroke insult.

## Discussion

We questioned whether acute administration of n-3 TG would provide neuroprotection in neonatal H/I brain injury and sought to determine the specificity of different TG in protecting brain tissue. Cerebral ischemic injury (stroke) is a serious problem in all periods of the life cycle. We chose to focus on the neonatal mouse model, not only because stroke is an important problem in the neonatal period in humans, but also because underlying mechanisms of H/I are well studied in this model [17], [26]–[28]. There was significant neuroprotection by n-3 TG in this model. In all experiments, ischemic injury is localized to the hemisphere ipsilateral to ligation and was characterized by infarction and neuronal tissue loss. Also, n-3 treatment decreased brain injury by attenuating tissue injury in multiple regions of the brain. Somewhat surprisingly, we found that these neuroprotective effects were limited to TG-enriched only in DHA, but not EPA. Also, neuroprotection was evident when n-3 TG were injected 2 hr after the H/I injury.

It is well recognized that TG composition of n-3 lipid emulsions affects blood clearance catabolism and tissue uptake [19], [29]. This study demonstrated that n-3-rich TG emulsions are also systemically absorbed after i.p. injection, and can substantially raise blood TG levels within short time periods, which in our experiment peaked at 1.5 hr post injection. The emulsion was efficiently catabolized as evidenced from blood TG levels dropping to baseline levels within 3 hr. Still, experiments with more time points after n-3 TG injection would be informative.

We first questioned whether acute administration of n-3 TG would provide neuroprotection at fixed doses of n-3 TG in our model of 10-day-old mice. The experimental doses were consistent with doses of intravenous lipid emulsions in human neonates at 1–1.5 g total TG/kg/day (0.5–0.75 g n-3 TG/kg). We initially demonstrated that n-3 TG, when administered in a “preventive” approach (pre-H/I treatment protocol), attenuated cerebral injury by reducing cerebral infarction in keeping with results from Berman [27] and Pan [30] in different neonatal and adult rat DHA protocols using pre-event i.p. administration, indicating that pretreatment with n-3 TG confers positive neuroprotection from brain H/I. Our preliminary data in pre-H/I treatment protocols also showed similar levels of neuroprotection by n-3 TG in adult mouse and rat brain H/I injury models [28]. The effects of the age disparity between the models utilized, such as varied substrate utilization in the developing brain, and potential differences in cerebral vasculature [31], [32], are differences that could have an effect on mechanisms of neuroprotection. However, even with these potential differences we still observed the neuroprotective effect from n-3 treatment. In each case, ischemic injury was localized to the right hemisphere ipsilateral to ligation and was characterized by infarction, neuronal tissue loss and an increase in degenerating neurons. n-3 TG treatment decreased brain injury by attenuating tissue injury in multiple regions of the right brain [28].

We demonstrated that when administered in a more “therapeutic” approach i.e., in post-H/I only treatment protocols, n-3 TG attenuated cerebral injury by reducing cerebral infarction size, indicating that post-H/I treatment with n-3 TG confers positive neuroprotection from brain H/I. Another question is whether neuroprotective effects seen here are a result of the combined effects of EPA and DHA contained in the emulsion, or related more to one of these FA; we used laboratory-made Tri-DHA or Tri-EPA emulsions to address this. We found that acute post-H/I treatment with Tri-DHA, at a lower dose (0.1 g n-3 TG/kg/dose) and higher dose (0.375 g n-3 TG/kg/dose), both resulted in decreased cerebral infarct volumes, with protection most marked in the subcortical regions of the brain tissue. Interestingly, no protection was observed after Tri-EPA injection (Figure 4). In studies of the long-term consequences of the H/I insult, we found that Tri-DHA showed cerebral neuroprotection 8 wk after injury with much less cerebral volume loss.

In clinical studies, it was shown that DHA and EPA have TG lowering effects [33]. However, in animal models of cerebrovascular diseases such as stroke and myocardial infarction, DHA appears to confer more promising organ protection than does EPA [30], [34], [35]. Underlying mechanisms for these differences need to be determined. Our preliminary data [28] indicates that neuroprotective effects of the n-3 TG emulsions in our model are likely related, in part, to NPD1, a potent derivative docosanoid of DHA. Endogenous response to H/I is also associated with an elevation of NPD1 in saline treated adult mice [36]. These results demonstrate that ischemic injury activates the cascade of events that lead to increased production of NPD1. In our preliminary studies in adult mice [28], post-ischemic elevation of NPD1 was significantly enhanced by the administration of an n-3 rich TG emulsion containing DHA and EPA (data not shown). As a result, we suggest that the n-3 TG emulsions might add to the existing reservoir of n-3 FA, in brain or other organs and that the enzyme systems activated by ischemia in H/I mice catalyze two pools of DHA substrate - the endogenous DHA liberated from the phospholipid membrane and exogenous DHA from the n-3 TG emulsion. AA derived lipid messengers 12(S) HETE, 15(S) HETE, and lipoxin A4 were also measured in whole brain from saline treated adult mice and n-3 TG treated mice (data not shown). These latter preliminary measurements were made because in addition to liberation of DHA from the phospholipid membrane in response to H/I, AA is also liberated by PLA_2_ and could have potentially led to elevated levels of its metabolites. However, there were no differences between groups for any of these metabolites (data now shown), in support of the hypothesis that the increased NPD1 levels are likely related to the DHA in the administered n-3 TG emulsion. As NPD1 attenuates classical features of inflammation, and protects cells from oxidative stress-induced apoptosis [37], it is likely that the neuroprotective effects of n-3 FA in our neonatal models also involve NPD1’s effects on these pathways. Nevertheless, to gain deeper understanding of the effects of NPD1 in our model it will be important to study the downstream mechanisms affected by NPD1 such as decreased polymorphonuclear leukocytes infiltration, and effects on apoptotic gene signaling as NPD1 has been shown to inhibit leukocyte infiltration [38] and apoptosis [39] in other models.

The timing of the administration of n-3 TG may be of significance as the various mechanisms involved in H/I injury occur over a wide time span, from the initial minutes to several days following the ischemic insult [3]. We examined whether DHA could still confer neuroprotection in H/I brain injury in the case of delayed treatment after H/I insult. In our n-3 TG dose-response experiment, both the low dose and the high dose of DHA were neuroprotective in reducing infarct volume when administered immediately after H/I insult. Again, the neuroprotective effect of DHA was demonstrated mostly in the subcortical region (penumbra area) by very substantial reductions in cerebral infarct volume when first administered at 0 h, 1 h and 2 hr after injury, respectively. Our findings are consistent with a study of Belayev [40] using an adult rat model where delayed treatment with DHA conferred neuroprotection. Their findings showed that DHA treatment could be delayed up to 5 hr after the stroke onset, different than our finding in neonatal mice where we did not observe neuroprotection 4 hr after H/I. This may be related to differences in the pathways of stroke progression in adult vs. neonate rodents, as well as possible differences in the mechanisms of neuroprotection.

Of special note, differences in metabolic rate may play a major role in defining the rapidity of apoptotic responses in mice vs. humans. Theoretical estimation indicates that the energy expenditure per kg in different size mammals is inversely proportional to body weight [41]. On this basis, it was assumed that metabolic rate and energy utilization per kg body weight in mice is 7 times higher in mice than in humans [42], [43]. Therefore, two hours in delay treatment in our protocol may represent substantially longer time “windows” in terms of new therapeutic approaches for stroke in humans with n-3 TG emulsions.

The aim of some therapeutic treatments, in large part, remains focused on reperfusion to the area of infarct plus symptom relief [44]–[47]. An initial insult in ischemic stroke is a reduction in CBF [5], [48]. The duration and intensity of the blood flow deficit are associated with the severity of brain damage. We measured CBF during hypoxia to determine the effect of n-3 FA treatment in neonatal mice. However, we found that there were no differences in CBF between n-3 TG treated and saline treated neonatal mice after H/I, indicating that other mechanisms are involved in our experiments.

Also, it is very unlikely that the extra energy supply of n-3 TG emulsions had any effects on neuroprotection. n-3 TG and n-6 TG emulsions injected had similar caloric densities, but as seen in Figure 3, n-3 TG provided neuroprotection but n-6 TG did not.

Our results provide new insights into the potential of employing n-3 TG, specifically DHA as a unique long chain FA aiding in neonatal H/I brain injury. A number of pathways are likely involved in n-3 TG neuroprotection [5]. For example, chronic administration of DHA resulted in increases of DHA levels in brain mitochondria. Increasing DHA might induce anti-apoptotic Bcl-2 proteins and inhibit apoptotic Bcl-2 proteins as well as improve mitochondrial capacity to handle excessive intracellular Ca^2+^ concentrations after H/I insult [5], [30].

Also, reperfusion injury after H/I is now well-known as a cause of the generation of oxidative radicals and the release of pro-apoptotic proteins contributing to the cellular damage [49]. Interactions among multiple interrelated mechanisms need to be addressed in order to improve cell survival, thereby reducing morbidity and mortality. The data from Belayev [40] in a post-stroke protocol indicate that DHA treatment not only protects both neurons and astrocytes, but also attenuates microglia activation, which leads to the initiation of apoptotic cascades. In ongoing and in preliminary experiments after H/I in neonatal mice we have found that injection of n-3 TG lipid emulsion reduced reactive oxygen species (ROS) production in whole brain, and this was associated with markedly reduced emission of ROS from mitochondria assessed at 30–60 min after initiation of reperfusion [50].

n-3 FA have been found to be safe and effective in the treatment of other neurological injuries including spinal cord injury [51]–[53]. Of interest both EPA and DHA showed neuroprotection after spinal cord injury [51], [52]. Reasons for lack of effect of Tri-EPA in our studies remain to be determined.

Our findings suggest a need for further studies to determine if acute injection of n-3 TG could be neuroprotective after stroke injury in humans. We also hypothesize that n-3 FA in TG, specifically DHA, will prove to be a “novel” and important therapy to treat stroke and could decrease mortality and increase long-term functional recovery after stroke in humans of different ages.

## Supporting Information

Table S1 **Fatty acyl composition of lipid emulsions^1^ (%).**
(DOCX)
