# Supplementary material for: N-3 Fatty Acid Rich Triglyceride Emulsions Are Neuroprotective after Cerebral Hypoxic-Ischemic Injury in Neonatal Mice
Source: PLoS One. 2013 Feb 20;8(2):e56233. doi: 10.1371/journal.pone.0056233 (PMC3577805; doi:10.1371/journal.pone.0056233)
Supplement: Table S1 — Fatty acyl composition of lipid emulsions1 (%). (DOCX) [file pone.0056233.s001.docx]

| **Supplemental Table 1.** Fatty acyl composition of lipid emulsions^1^ (%) | | |
| --- | --- | --- |
|  | **n-3 TG** | **n-6 TG** |
| **Source** |  | g/100mL |
| Soybean oil | - | 20 |
| Fish oil | 10 | - |
| Egg phosphatidylcholine | 1.2 | 1.2 |
| Glycerol | 2.5 | 2.25 |
| **FA (% of total FA)** |  | % |
| Palmitic acid (C16:0) | 2.5-10 | 7-14 |
| Stearic acid (C18:0) | 0.5-2 | 1.4-5.5 |
| Oleic acid (C18:1n-9) | 6-13 | 19-30 |
| Linoleic acid (C18:2n-6) | 1-7 | 44-62 |
| Arachidonic acid (C20:4n-6) | 1-4 | <0.5 |
| α-linolenic acid (C18:3n-3) | 2 | 4-11 |
| Eicosapentaenoic acid  (C20:5n-3) | 12.5-28.2 | - |
| Docosahexaenoic acid  (C22:6n-3) | 14.4-30.9 | - |
| ^1^Data provided by Fresenius Kabi AG; FA, Fatty acids. | | |
